# Heating influence on hierarchical structures fabricated by direct laser interference patterning

**DOI:** 10.1038/s41598-022-22368-w

**Published:** 2022-10-22

**Authors:** Nikolai Schröder, Fabian Nyenhuis, Robert Baumann, Lucinda Mulko, Thomas Kiedrowski, Johannes Albert L’huillier, Andrés Fabián Lasagni

**Affiliations:** 1 Institut für Fertigungstechnik, Technische Universität Dresden, George-Bähr-Strasse 3c, 01069 Dresden, Germany; 2 Bosch Research, Robert Bosch GmbH, Postbox 30 02 40, 70442 Stuttgart, Germany; 3 Photonik-Zentrum Kaiserslautern e.V. and Research Center OPTIMAS, Technische Universität Kaiserslautern, Kohlenhof Strasse 10, 67633 Kaiserslautern, Germany; 4 Fraunhofer-Institut für Werkstoff- und Strahltechnik (IWS), Winterbergstrasse 28, 01277 Dresden, Germany

**Keywords:** Metals and alloys, Ultrafast lasers, Mechanical engineering

## Abstract

The combination of direct laser interference patterning (DLIP) with laser-induced periodic surface structures (LIPSS) enables the fabrication of functional surfaces reported for a wide spectrum of materials. The process throughput is usually increased by applying higher average laser powers. However, this causes heat accumulation impacting the roughness and shape of produced surface patterns. Consequently, the effect of substrate temperature on the topography of fabricated features requires detailed investigations. In this study, steel surfaces were structured with line-like patterns by ps-DLIP at 532 nm. To investigate the influence of substrate temperature on the resulting topography, a heating plate was used to adjust the temperature. Heating to 250 $^{\circ }$C led to a significant reduction of the produced structure depths, from 2.33 to 1.06 µm. The reduction is associated with the appearance of a different LIPSS type, depending on the grain orientation of the substrates and laser-induced superficial oxidation. This study revealed a strong effect of substrate temperature, which is also to be expected when heat accumulation effects arise from processing surfaces at high average laser power.

## Introduction

Surface engineering methods based on ultrashort pulsed laser irradiation are a cutting-edge topic in the scientific and industrial sectors as they enable the improvement of surface properties of paramount relevant materials^1^. Specifically, tailor-made laser-induced surface functionalities are at the state-of-the-art for a wide range of industrial sectors and application scenarios^1–3^. For instance, Vercillo et al. demonstrated ice repellency on a titanium alloy for aerospace applications based on laser-induced superhydrophobicity^4^. Epperlein et. al reported that nano-scale features obtained with laser surface structuring could influence biofilm growth or its inhibition on steel samples^5^. Furthermore, improved optical properties of organic solar cells were achieved by Guay et al.^6^ Hence, laser structuring techniques are able to produce structural elements with high resolution due to the controlled ablation of surface material^1^.

A suitable laser structuring method for achieving such periodic surface structures is direct laser interference patterning (DLIP). DLIP relies on the near-surface interference of two or more laser beams creating a patterned surface with features in the micro- and nano-meter range^7^. Depending on the number and polarization of the laser beams, DLIP is capable of designing and producing a variety of different topographical surface architectures. A promising approach is the combination of the DLIP structures with even smaller laser-induced periodic surface structures (LIPSS) and thus creating a surface topography with a complex structural hierarchy^8–12^. In nature, these hierarchical structures have already been shown to even reach higher performances compared to single-scale patterns^13^.

LIPSS features are subject to a self-amplifying process (positive feedback) based on an increasing near-surface modulation of the irradiated intensity distribution. This is due to an increasing nano-roughness with a growing number of applied laser pulses^14–16^. The modulation results mostly from interference of the irradiated wave with refracted and scattered wave components or with the electromagnetic field of surface plasmons^15,17–21^. The formation of LIPSS is also affected by the temporal spacing of the pulses^22,23^. In particular, when surfaces are processed at high throughput, higher average laser powers are mandatory. This usually requires the use of high repetition rates, i. e. in the MHz range. Therefore, short temporal distances between the laser pulses occur, leading to heat accumulation effect^23–26^. This effect results in an overall increase of the surface temperature which can significantly affect the structuring mechanism in laser-ablation processes.

In previous works, Rudenko et al. and Tsibidis et al. discussed convective structure formation mechanisms, which should become increasingly important with stronger heat accumulation^19,27^. In addition, Bauer et al. associated a critical quantity of heat accumulation with bumpy surface structures on the micrometer scale^24^. Despite such heat-induced structure formation processes, it is often assumed that process throughput can be enhanced by simply increasing the repetition rate^28^. Although this in turn cannot be achieved without a large increase in heat accumulation. Therefore, process strategies providing hierarchical topographies are probably not transferable to higher repetition rates without changes in process dynamics and structure formation^9,12^. In this frame, it is of great relevance to investigate how the substrate temperature affects the DLIP formation process, especially when fabricating hierarchical surface patterns due to the simultaneous formation of LIPSS.

The aim of the present study is to evaluate the influence of substrate temperature on the resulting surface topography when using DLIP treatment on stainless steel with ps pulses. During the laser process, a heating plate is used to adjust the substrate temperature of the samples up to 250 $^\circ$C. The obtained surface structures are characterized using confocal microscopy, scanning electron microscopy and energy-dispersive X-ray spectroscopy.

## Results

### Surface topography examination using scanning electron microscopy and Fourier transform analysis

**Figure 1:**
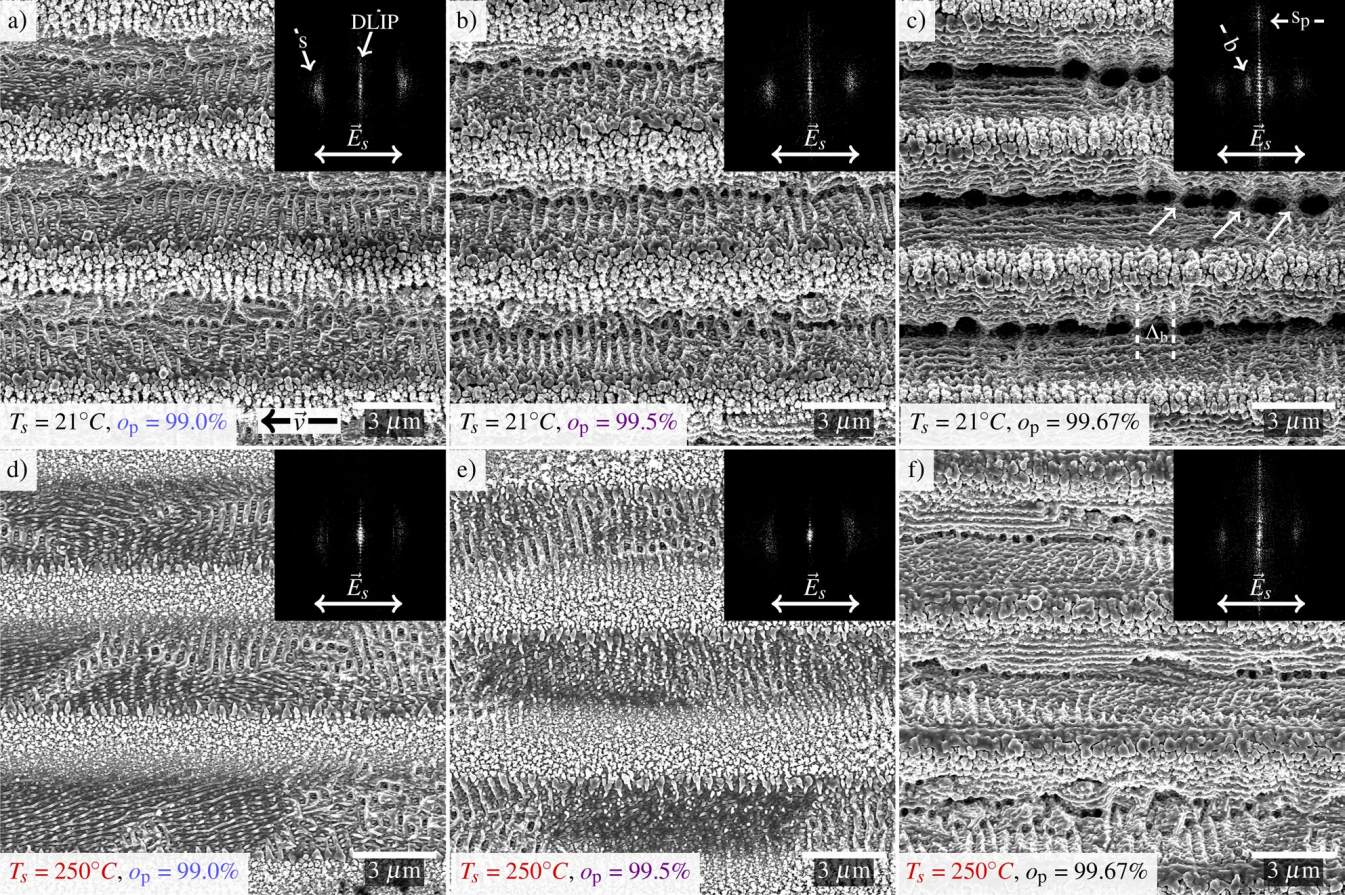
SEM images of the surface structures generated using DLIP. The peak fluence was $\Phi _\mathrm {p}$ = 0.5 J/cm$^2$ (for Gaussian equivalent without interference) and the repetition rate *f* = 200 kHz. The sample temperatures, polarization, and overlaps are depicted in the images. The movement of the positioning stage is marked by a black arrow in (**a**). The black insets show the corresponding FFT obtained from a 37.25$\times$37.25 µm SEM image (shown till a wavevector of $\vec {k}\cdot (2\pi )^{-1}$ = 200 nm). The process parameters are indicated in each figure.

In the first set of experiments, the steel substrates were treated using a two-beam DLIP configuration with a spatial period of 4.5 µm and a substrate temperature $T_{\mathrm {s}}$ of 21 $^{\circ }$C, hereinafter referred to as “non-heated” surfaces. In this case, the pulse-to-pulse overlap $o_{\mathrm {p}}$ represents the distance between two pulses as a function of the spot size. It was varied from 99.0 % (100 pluses per position) to 99.67 % (300 pulses per position). In all cases, a peak fluence $\Phi _\mathrm {p}$= 0.5 J/cm$^2$ (for Gaussian equivalent without interference) and a repetition rate *f* = 200 kHz was applied. The laser beam polarization was oriented parallel to the movement of the positioning stage (Fig. 1a)), which is parallel to the direction of the line-like geometry produced by the two-beam interference pattern. Representative scanning electron microscope (SEM) images of the produced structures are shown in Fig. 1a–c. To support the analysis of the SEM pictures in terms of the topography, the Fourier Transform (FFT, shown as dark inset) of the evaluated structure is performed. In all cases, the produced DLIP geometry is visible, with a spatial period of 4.5 µm.

For the case of $o_{\mathrm {p}}$ = 99.0 %, in the darker areas of Fig. 1 a, which correspond to the interference maxima positions, trenches containing smaller parallel structures can be observed. These alternate with brighter stripes covered with nano-particle-like topography. As the parallel structures in between the trenches appear perpendicular to the laser beam polarization and exhibit a period $\Lambda _{\mathrm {LSFL-I}}$ of 418$\pm 65$ nm, which is just below the used laser wavelength $\lambda$ (532 nm), can be referred to as low spatial frequency LIPSS (LSFL-I)^15,18^. The LSFL-I cause so-called type-s signal, “s” for scattering^15,20^, in the FFT. Thus, this signal is perpendicular to the strong central vertical features, which in turn result from the DLIP structure ($\Lambda _{\mathrm {DLIP}}$ $\approx$ 4.5 µm). The signal generated by the line-like structures of the DLIP pattern in the FFT images is denoted as “type-DLIP”.

By further examining Fig. 1, it can be seen that as the overlap $o_{\mathrm {p}}$ increases, the type-s signal is directionally more concentrated along the x-axis of the FFT. The remaining LSFL-I tend to be more parallel. Furthermore, the relative intensity of the type-s signal decreases, and the type-DLIP signal increases. This is due to the increasingly pronounced trenches with higher overlap. Moreover, the signal on the abscissa between type-s and the center must result from a structure with the same orientation as LSFL-I, but with a larger period ($\Lambda _\mathrm {b}$ $\approx$ 1.4 ± 0.2 µm), as shown in Fig. 1c). Consequently, their formation is presumed to be the pattern of the pits in the center of the trenches. In the high-frequency range (large wavenumbers) of the ordinate also a new feature appears. The signal originated from parallel ripples on the slopes of the trenches, which most likely results from the interference of incident and the forward-reflected light at the slopes^9,14^. In the following, these ripples were denoted by LSFL$_\mathrm {edge}$ and their signal by type-s$_{\mathrm {p}}$.

In further experiments, the temperature of the sample was adjusted to 250 $^{\circ }$C, in the following so-called “heated” surfaces. The structuring was performed according to the same processing strategy as the experiments mentioned in the previous section (Fig. 1a–c). The SEM images, illustrated in Fig. 1d–f, depict the resulting topography. Heating of the samples up to 250 $^{\circ }$C resulted in increased emergence of LSFL, orientated parallel to the laser polarization. These structures can be characterized as LSFL-II and exhibit a spatial period $\Lambda _\mathrm {LSFL-II}$ of 247 ± 35 nm. The LSFL-II signal is not shown in the FFTs due to the high frequency of the pattern. With increasing $o_{\mathrm {p}}$ from 99.0 to 99.67$\%$ (Fig. 1d–e), the width of the regions with bright stripes increases, which causes the DLIP signal to occur at higher wavenumbers (lower frequency) and consequently to be shifted to the center of the FFT. The rows of pits in Fig. 1d are probably precursors of the so-called grooves that form vertically from LSFL-I^22,27^. In addition, LSFL-II seems to become shorter and with an irregular appearance. It is likewise noticed that the average size of the bright stripes with nano-particle-like topography is smaller for this case. Moreover, the size distribution of these nano-particles appears to be less dispersed (or results in less agglomerated particles) than in the non-heated case. This can be qualitatively assessed by comparing Fig. 1a,d or b,e, respectively.

With a further increase of the overlap $o_{\mathrm {p}}$ to 99.67 % (Fig. 1f), a distinct topographic gradually arises due to increasingly pronounced trenches. However, these trenches appear less orderly and less deep than in Fig. 1c. This is qualitatively noticeable by the lower contrast between bright and dark areas in the images. These findings are further supported by the weaker and more diffuse ordinate signal of the FFT in Fig. 1f compared to the FFT in c. The shallower trenches, later confirmed by confocal microscopy, in the heated case are also noticeable when comparing Fig 1b,e.

**Figure 2:**
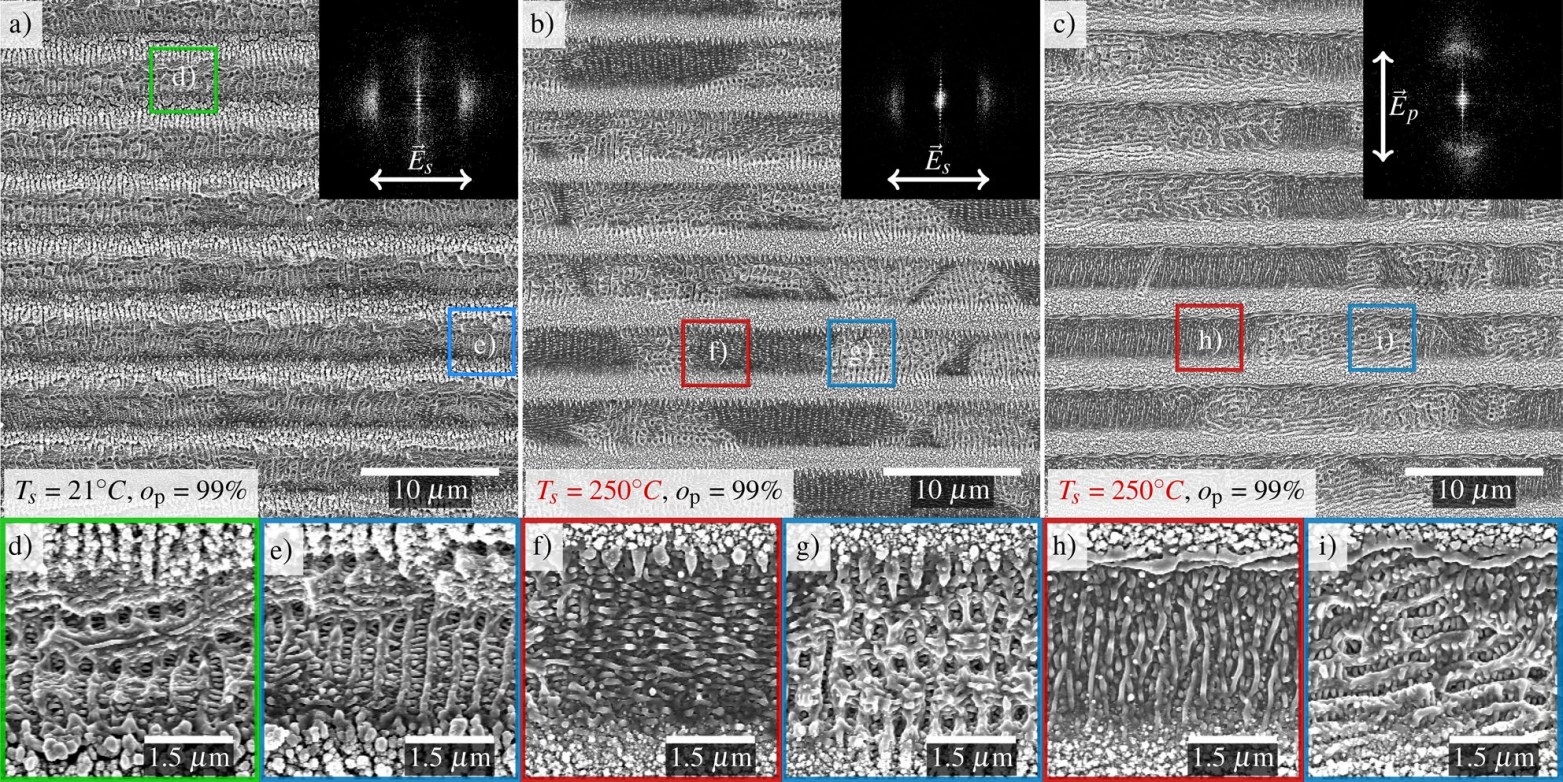
SEM images of the surface structures generated using DLIP. The process parameters were the same as those set in Fig. 1. The sample temperature $T_s$, polarization, and pulse overlap $o_\mathrm {p}$ are depicted in the images. The black insets show again the corresponding Fourier transforms. The images in (**d**)–(**i**) are enlargements of the marked areas in (**a**)–(**c**).

In addition to the previous experiments, the polarization of the laser beam was rotated by 90$^{\circ }$, leading to an orientation of the polarization vertical to the movement of the positioning stage. Figure 2a–c shows the early stage of structure formation at $o_{\mathrm {p}}$ = 99.0 % at the non-heated (a), heated (b), and heated with 90$^{\circ }$-rotated polarization (c) cases. For visualizing the nano-topography of the structures, the areas marked with colored squares are shown as enlargements in Fig. 2d–i.

As it can be seen in this case, the structures inside the darker regions of Fig. 2b,c are polarization-sensitive and hence denoted as LSFL-II^14,20,29,30^. Noteworthy, the orientation of the LSFL-I was also rotated (Fig. 2g,i), which can be noted in the orientation of the type-s signal in the corresponding FFTs. The bandwidth of the LSFL-I period appears larger as well as its range is shifted to smaller periods in Fig. 2c compared to b, as indicated by a more broadly distributed type-s signal. In summary, the following spatial periods of LSFL at different heating temperatures can be observed on the samples: $\Lambda _{\mathrm {LSFL-I}}$ = 418$\pm 65$ nm at 21 $^{\circ }$C (Fig. 2a), $\Lambda _{\mathrm {LSFL-I}}$ = 445$~\pm$ 67 nm and $\Lambda _{\mathrm {LSFL-II}}$ = 247 ± 35 nm at 250 $^{\circ }$C (Fig. 2b) for the s-polarization. In contrast, the spatial periods for the p-polarization and 250 $^{\circ }$C are $\Lambda _{\mathrm {LSFL-I}}$ = 390$\pm 55$ nm and $\Lambda _{\mathrm {LSFL-II}}$ = 265±35 nm (Fig. 2c).

Remarkably, the findings show that only by increasing the sample temperature, the surface topography switches between two extreme cases including (i) surfaces containing only LSFL-I features and (ii) areas covered by LSFL-II. Since the formation of this specific type of LIPSS on metallic surfaces are associated with a superficial oxide layer, an energy dispersive X-ray (EDX) analysis was performed. Table 1 summarizes the obtained results. Each assay is achieved by averaging at least four spectra at different surface positions on the processed sample. The measurements were performed at various sample temperatures $T_\mathrm {s}$ and different surface positions of the specimens, which contain unstructured or structured areas. The measured data also contain information about the deeper non-oxidized layers that are located just below the treated molten area but within the electron penetration depth of the EDX analysis. However, it should be noted that the capability of EDX to quantitatively determine oxygen contents is limited and that the values can therefore only provide a qualitative assessment here.

**Table 1:** Energy-dispersive X-ray analysis of stainless steel specimen at different sample temperatures $T_\mathrm {s}$ and surface positions. The errors are standard deviations.

Sample	$T_\mathrm {s}$ / $^\circ$C	C/at%	O/at%	Si/at%	Cr/at%	Fe/at%	Ni/at%
unprocessed	21	14.6 ± 2.1	–	0.8 ± 0.1	17.6 ± 0.4	60.8 ± 1.6	6.3 ± 0.3
unprocessed	250	18.5 ± 2.2	–	0.7 ± 0.1	16.6 ± 0.5	58.2 ± 1.6	6.0 ± 0.2
LSFL-I	21	8.7 ± 1.2	4.7 ± 0.6	0.1 ± 0.1	18.1 ± 0.3	61.7 ± 0.7	6.3 ± 0.1
LSFL-I	250	14.0 ± 0.6	7.2 ± 0.4	0.6 ± 0.1	16.2 ± 0.2	56.1 ± 0.7	5.8 ± 0.1
LSFL-II	250	13.7 ± 2.3	5.9 ± 0.5	0.7 ± 0.1	16.5 ± 0.4	57.2 ± 1.9	6.0 ± 0.2
LSFL-I & -II	250	13.9 ± 1.5	6.6 ± 0.5	0.6 ± 0.1	16.3 ± 0.3	56.7 ± 1.3	5.9 ± 0.2

The unprocessed areas on the samples for all working temperatures show no significant amount of oxygen. After the laser treatment, although in all cases, there is an increased amount of oxygen content^31^. The difference in the elemental compositions of the two unprocessed samples is as expected for commercial steel samples and the detected value of carbon is significant higher compared to the manufacturer data sheet of AISI 304 steel due to hydrocarbon contamination^32^.

Prior to the discussion of possible causes of the reduced ablation depth of the trenches and the change from LSFL-I to LSFL-II, the interpretation of the experiments is supported by quantitative analyses of the surface topography using the power spectral density (PSD) and height profiles.

**Figure 3:**
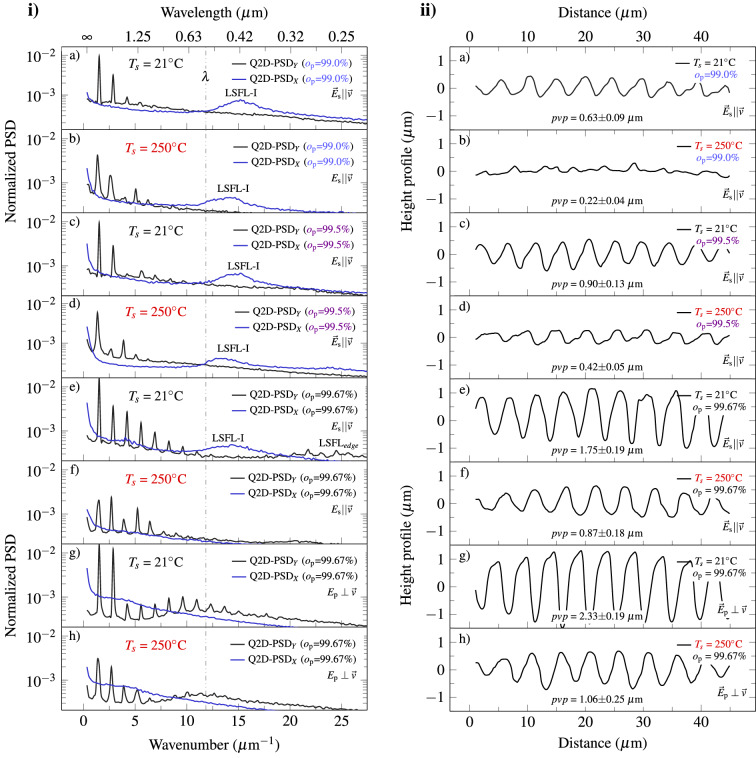
(**i**) Quasi-two-dimensional normalized power spectral density (Q2D-PSD) of surfaces shown as SEM images in Figs. 1 and 2. Since the PSDs are normalized, a decrease in the total signal is to be understood as an increase in the constant part (*k* $\le$ 0.7 µm$^{-1}$, not shown), i.e., smoothness. (**ii**) Corresponding mean height profiles of the surfaces. The sample temperature $T_s$, the overlap $o_{\mathrm {p}}$ and the orientation of the laser polarization *E* to the movement of the positioning stage $\vec {v}$ are depicted in all plots.

### Topography analysis using power spectral density and confocal microscopy

To quantify the impressions from the SEM images, averaged normalized power spectra were generated from at least three SEM images per parameter set, by averaging over all one-dimensional (1D) power spectral density (PSD) in x- or y-direction. The corresponding plots are shown in Fig. 3i and reveal the frequency shifts of signals and their relative contributions to the spectrum.

In Fig. 3ia,c,e, a growing trend in the DLIP-peaks around $k_{\mathrm {DLIP}}~=~2\pi$ (4.5 µm)$^{-1}$ = 1.4 µm$^{-1}$ or the corresponding higher harmonics with increasing overlap $o_{\mathrm {p}}$ is observed. The increment in the amplitude of the fundamental correlates with the stronger development of the DLIP structure. The amplitudes of higher harmonics rise with the steepness of the slopes. For a rectangular function as a limiting case, the largest number of frequencies would be required for approximation. Therefore, the peak around 1.4 µm$^{-1}$ in the PSD, as well as corresponding harmonics, may serve as a quality parameter for the shape of the trenches.

Instead, the PSD of the heated samples, shown in Fig. 3(i)b,d,f, depict weaker and broader peaks, and less signal in the associated harmonics. Moreover, in Fig. 3(i)f, a 2nd harmonic signal is observed that even exceeds the signal of the fundamentals. This reflects the more irregular and less pronounced DLIP structure of the heated samples (compared to $T_s$ = 21$^\circ$C). Another feature is that the resulting LSFL-I signal shifts to smaller wavenumbers (larger periods) with increasing overlap $o_{\mathrm {p}}$. This can be explained by the increase in the edge steepness of the DLIP pattern and the associated local increase in the angle of incidence^14,33^. Following this trend, the broadening of the LSFL-I signal could also be explained. In addition to the steep slopes, there are also flat areas at the bottom and over the ridges of the DLIP structure, allowing for a wider range of LSFL-I periods. For strongly absorbing materials, the period of LSFL-I is usually estimated as:

1 $$\begin{aligned} \Lambda _{\mathrm {LSFL-I,p}} \sim \frac{\lambda }{1\pm \sin {\theta }} \quad \quad \text {and} \quad \quad \Lambda _{\mathrm {LSFL-I,s}} \sim \frac{\lambda }{\cos {\theta }}, \end{aligned}$$

where $\theta$ is the angle of incidence and the subscripts s and p refer to the different polarizations^33^.

**Figure 4:**
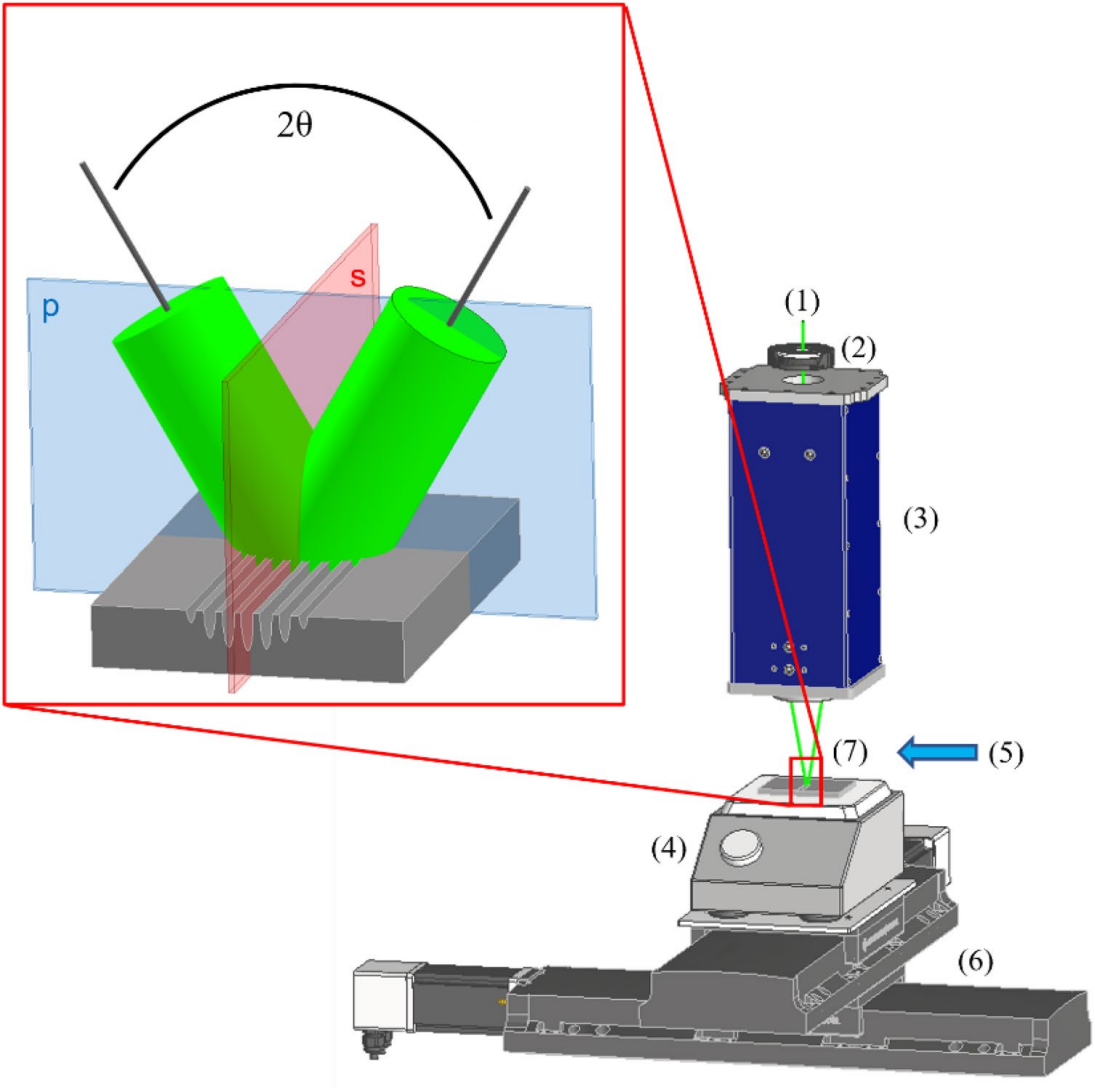
Illustrative diagram of the experimental setup of the two-beam-DLIP arrangement including (1) laser beam, (2) $\lambda$/2-plate, (3) DLIP head with specific optic configuration, (4) heat plate, (5) cross jet, (6) x,y-positioning stage and (7) stainless steel sample. Framed in red on the left, the two superimposed sub-beams at an angle $2\theta$ including the s- and p-polarization are exhibited producing a line-like structure on the sample.

It should be noted that the incidence plane of a DLIP setup is usually perpendicular to the movement of the positioning stage, as shown in Fig. 4 (see “Materials and Methods” section). Therefore, the s-polarization is usually parallel to the movement of the stage and the p-polarization is perpendicular to it. According to Eq. (1), a broadening and shifting of the LSFL-I signal towards smaller wavenumbers would be expected for s-polarization. This was caused by an increase in trench depth and thus an increase in $\theta$ and the angle range $\theta \pm \delta \theta$. This can be seen by comparing the LSFL-I peaks in Fig. 3ia,c,e.

In agreement with the results shown in Fig. 1c, the LSFL$_\mathrm {edge}$ are also visible in the corresponding PSD of Fig. 3ie. Figures 3ig,h show the PSD for p-polarization. The difference in DLIP peaks is even more pronounced between the heated and non-heated samples. In this case, the signals from the LSFL-I and the higher harmonics of the DLIP peaks overlap, increasing the signal around the laser wavelength.

To discuss the results in more detail, the structure depth of the line-like DLIP height profiles at different temperatures and pulse-to-pulse overlaps are displayed in Fig. 3ii. The vertical height profiles of surfaces were obtained by averaging over ten individual vertical height profiles around the center of the DLIP structure. For each applied temperature, the structure depth is increasing with higher pulse overlap. The profiles of the heated samples exhibit trenches with a mean peak-valley-peak (*pvp*) value of 0.87 µm for the s-polarization and 1.06 µm for the p-polarization. In comparison, the non-heated samples show a *pvp* of 1.75 µm and 2.33 µm for the s-polarization and p-polarization, respectively. The corresponding *pvp* are depicted within the height profiles of Fig. 3ii. Each mean *pvp* value is calculated by averaging eight single *pvp*.

In addition, height profiles for p-polarization, which are perpendicular to the movement of the positioning system and trenches, are shown in Fig. 3iig,h. The p-polarization orientation has a positive effect on the trench depth, as it leads to a slightly higher *pvp* of 2.33 µm compared to the s-polarization with a *pvp* of 1.75 µm. This, in turn, parallels the trenches and movement of the positioning stage system. This effect may be caused by smaller structures in the case of s-polarization compared to the case of p-polarization (see Fig. 2f,h) which is discussed further in the next section.

## Discussion

The discussion aims to interpret the decrease in trench depth in the case of heated samples from the change (LSFL-I to LSFL-II) in the dominant LIPSS class. Therefore, the following questions are addressed:

(i) Why do the heated samples exhibit lower trench depth?
(ii) Why are more LSFL-II predominantly formed on heated samples?
(iii) Why do the LSFL-II appear in clusters?

To address the first question, the mechanisms that cause reduced ablation must be considered. For a single pulse with perpendicular incidence, the ablation depth can be described by:

2 $$\begin{aligned} \Delta z = \delta _{\mathrm {E}} \cdot \mathrm {ln}(\Phi /\Phi _{\mathrm {th}}), \end{aligned}$$

where $\delta _{\mathrm {E}}$ is the energy penetration depth and $\Phi$ and $\Phi _{\mathrm {th}}$ are the absorbed fluence and fluence threshold for ablation, respectively^34^.

Mathematically, energy penetration depth has a multiplicative effect on ablation depth, while changes in fluences have a logarithmic effect. Therefore, changes in fluence do not affect $\Delta z$ strongly, as long as $\Phi ~\gg ~\Phi _{\mathrm {th}}$. However, strong oxidation, e.g., by forming chromium oxides, results in stronger Cr-O bonds compared to Cr-Cr bonds^35^, thereby increasing the ablation threshold. Thus, $\Phi ~\gg ~\Phi _{\mathrm {th}}$ is not well satisfied anymore, leading to a rapid decrease of ablation depth with decreasing fluence. In addition, a correlation between the extent of oxidation and the LSFL-II period is known, which is explained by the changed optical properties of the surface caused by the nanostructures themselves and the surface oxidation^30,35^. Consequently, the exact near-surface distribution of absorbed fluence $\Phi$ results from complex dynamics of mutually influencing structure period and oxide layer thickness^30^. Depending on the period, nanostructures strongly influence the absorbed fluence distribution due to substantial field enhancement, surface plasmon excitation, extraordinary optical transmission or scattering^17,19–21^. As a result, $\Phi$ is highly inhomogeneous near the surface, and $\delta _{E}$ most likely can no longer be determined with Lambert-Beer using a single absorption coefficient $\alpha = \delta _{\mathrm {opt}}^{-1} \approx \delta _{\mathrm {E}}^{-1}$ for the entire near-surface volume. Since the oxide film thickness strongly depends on the solidification time^26^, the named effects depend on the sample temperature. A light microscopic image shown in Fig. S1 in the supplemental material indicates altered optical properties.

These influences partly explain the shallower trench depth in the case of the smaller surface structures in Figs 1d,e and 2b,c and 3(ii)b,d,f, respectively.

LSFL-II are known to form on semiconductors, dielectrics, and materials that are prone to oxidation^14,29,30,36,37^. In the latter case, the thickness of a superficial oxide layer is particularly crucial^30^. The performed EDX analysis shows a formation of surface oxides on the structured surfaces. In this way, for the non-heated samples, it appears that the surrounding oxygen contributes partially to the formation of gaseous species and partially to the formation of surface oxides. Both phenomena contribute significantly to the process. In contrast, for the heated samples, probably due to a beneficial kinetic effect, the formation of metal oxides in various oxidation states (SiO$_{\mathrm {2}}$, Cr$_{\mathrm {n}}$O$_{\mathrm {m}}$, Fe$_{\mathrm {n}}$O$_{\mathrm {m}}$, NiO, etc.) is significantly favored^38^. In addition to the required oxide layer, the presence of a sub-wavelength roughness, which is mostly high spatial frequency LIPSS (HSFL), is essentially required to form the necessary sub-wavelength intensity pattern (type-d)^14,30^. The final intensity pattern of LSFL-II is formed as a function of the amplitude of HSFL and the thickness of the oxide layer. The reason for this pattern is the far-field interference of the light scattered at the HSFL and the light refracted into the material and propagating inside the superficial dielectric material^20,29,30^. Evidence for the prior presence of HSFL is provided by SEM images of the edge of the surface pattern in Fig. S2 of the supplementary material section. This outer region undergoes the low fluence of the periphery of the intensity profile, enabling HSFL formation. Due to the symmetry of the intensity profile, this effect also occurs along the scan direction.

Heating the samples influences the LSFL-II formation process in several ways. On the one hand, increasing the sample temperature $T_\mathrm {s}$ affects the solidification and cooling rate significantly more than the thickness of the melted layer^26^. Consequently, the liquid interface of heated samples is exposed to the surrounding oxygen for a longer time. In addition, the delay in solidification allows the development of complex convection processes increasing the mixing of oxygen and oxides with the liquid steel^26^. This may be demonstrated by comparing the thickness of an oxide layer formed solely by diffusion ($\Lambda _\mathrm {diff}=\sqrt{D~\times ~t_\mathrm {s}}~\le ~15$ nm) for a corresponding solidification time of $t_\mathrm {s}~\le ~200$ ns and diffusion coefficient $D~\le$ 10$^{-5}$ cm$^2$/s) with the significantly higher thicknesses observed or required in LSFL-II formation^30^. On the other hand, heating also affects HSFL formation and thus the required scattering objects for the type-d intensity pattern that causes LSFL-II. Exposing nanovoids trapped beneath the surface demonstrated their involvement in the formation of HSFL^39^. These defects likely represent the electromagnetic origin of HSFL, due to the required high-frequency periodic intensity patterns^14,17,19,29^. In addition, the intensity pattern produced by these is more uniform, with a high number of nanovoids^19^. Consequently, the reasons for an increased occurrence of HSFL might be explainable through changes in the dynamics of crystal defects with rising $T_\mathrm {s}$.

Recently, it was demonstrated for silicon that the cooling rate is the critical parameter for the supersaturation of self-interstitials and thus, for the formation of dislocations by point defect accumulation^40,41^. Molecular dynamics simulations of pure metals indicate supersaturation of vacancies during rapid re-crystallization and consequently, a similar behavior for vacancies accumulation in metals^42–44^. Also, recent experimental studies for Ag focus on possible formation mechanisms of voids and clusters through the accumulation of point defects^45^. Consequently, increasing sample temperature $T_\mathrm {s}$ and thus decreasing the cooling rate may affect the formation of voids, which are the seeds for HSFL.

If vacancies are the required precursor to voids and thus HSFL, the sample temperature $T_s$ should have two effects. On the one hand, $T_s$ affects the re-crystallization rate and thus the point defect concentration (vacancy concentration) in the re-grown crystal. On the other hand, it also influences the cooling rate after solidification and thus the point defect diffusion within the crystal^40,41^. Furthermore, the solidification rate depends on the crystal orientation and is therefore strongly anisotropic, as well as the diffusion of point defects^42,43^. Following this premise, the light-matter interaction becomes anisotropic due to the anisotropic material response, which in turn enhances this deterministic periodic energy deposition. For polycrystalline materials, this behavior is likely to be limited to the dimensions of one grain. Indeed, grain orientation-dependent formation of LIPSS has already been demonstrated^46,47^. Therefore, the influence of the specimen temperature $T_s$ on crystallization rate is probably less strong than the influence of the grain orientations. Thus, different crystal orientations of different grains provide potential explanations for an increased occurrence of voids and thus the clustered occurrence of HSFL or LSFL-II, respectively.

To clarify the first indications for this hypothesis, an unprocessed sample was etched to reveal the grain formation close to the surface. The grains are compared in Fig. S3, which is shown in the supplementary material. Furthermore, the LSFL-I and LSFL-II appear group-wise on the heated sample. The dimension and the geometry of these clusters correspond to the size of the grains.

In addition, HSFL occur only in a narrow range of low fluence due to their convective origin^19,29,48^. Therefore, in the experiments, it is likely to occur only at the periphery of the beam profile. Consequently, the HSFL form on a non-oxidized or slightly oxidized surface, as it becomes evident by comparing the oxide fraction of the processed and unprocessed samples (see Table reftab:example). This reinforces the premise that the oxide layer is mainly laser-induced.

Given that the formation of LIPSS is generally a function of pulse number due to an inter-pulse feedback^14^, the HSFL may be replaced by larger structures as pulse overlap increases^19^. Less regular HSFL lead to a less regular intensity pattern (type-d) required for LSFL-II formation. Thus, as the overlap $o_\mathrm {p}$ increases (see Fig. 1 from d–e), the regularity of LSFL-II decreases.

## Conclusion

In this study, the influence of substrate temperature on the topography of laser-structured stainless steel surfaces processed with DLIP was investigated. It was found that heating of the substrate temperature from 21 to 250 $^\circ$C led to a decrease in the ablation depth from 1.75 to 0.87 µm at s-polarization and from 2.33 to 1.06 µm at p-polarization. This decrease was attributed to a change in LIPSS type from LSFL-I to LSFL-II, which is associated with a laser-induced superficial oxide layer at higher sample temperatures. In addition, LSFL-II have the potential to increase the threshold fluence resulting from increased oxidation. It is suggested that in this process regime, with high pulse overlap, moderate fluences, and moderate repetition rates, the occurrence of LSFL-II is also determined by changes in dislocation dynamics caused by the heating of the samples. It is hypothesized that the clustering of LSFL-II is due to the grain orientation-dependent nanovoid formation leading to HSFL as a precursor of LSFL-II. Moreover, the effect of polarization orientation on the structure period and the bandwidth of structure periods were investigated. It turned out that p-polarization is more effective for DLIP processes in terms of ablation depth. Overall, the present study reveals a set of process parameter to control and optimize the ablation depth of DLIP and to thus create tailor-made surface patterns. Finally, the change from LSFL-I to LSFL-II caused exclusively by heating is also expected for a slight increase in repetition rate at constant pulse overlap due to increased heat accumulation^24^. All these aspects are relevant for upcoming challenges for scaling-up DLIP processes, e.g. by using polygon scanner systems^49^. To minimize the resulting heat accumulation, the following strategy could be followed: keeping the scanning speed of the polygon scanner as high as possible while utilizing larger spot sizes of the laser beam orthogonal to the scanning direction and using the optimal ablation fluence^28^. Furthermore, these insights enable the creation of a complex hierarchical topography for advanced surface functionalization using DLIP.

## Material and methods

Electropolished stainless steel sheets (X5CrNi18-10, 1.4301, AISI 304) with a thickness of 0.8 mm were used in this study. To remove any impurities from the surfaces, the samples were gently rinsed with ethanol (Ethanol absolute $\ge$ 99.9 %) before laser processing.

The DLIP setup is illustrated in Fig. 4. The samples were structured using a DLIP system equipped with an ultra-short pulsed laser source with 12 ps pulse duration, 532 nm wavelength and a maximum repetition rate of 50 MHz. The spatial energy distribution of the beam was Gaussian. A custom-designed optic arrangement provided a two-beam interference configuration that produces line-like structures on the samples. A lens with a focal length of 100 mm superimposed the two laser sub-beams on the surface at a fixed angle of 6.8$^\circ$ to achieve a spatial period of approximately 4.5 µm. Further information about the experimental setup can be found elsewhere^50^.

The samples were placed on a heating plate that settle at a specific temperature prior to the laser processing. The temperature of the heating plate was set at 21 and 250 $^\circ$C. A cross jet compressed air flow combined with an extraction unit was used during all experiments to prevent dust depositions on the optics. A x,y-stage system was installed to position the sample during the structuring process.

The speed of the positioning stage system was varied between 66 and 200 mm/s in order to obtain pulse-to-pulse overlap from 99.0 to 99.67 $\%$, respectively. In all cases, the repetition rate was fixed to a value of 200 kHz with an average power of 4 W, resulting in a pulse energy of 20 µJ. The used beam diameter in the DLIP experiments was approximately 100 µm, obtaining a peak laser fluence of 0.5 J/cm$^{2}$. The total energy deposited per unit area represents the peak cumulated fluence and it corresponds to 50 J/cm$^2$ for $o_{\mathrm {p}}$ = 99.0 $\%$, 100 J/cm$^2$ for $o_{\mathrm {p}}$ = 99.5 $\%$, and 150 J/cm$^2$ for $o_{\mathrm {p}}$ = 99.67 $\%$. The polarization of the laser beam was changed using a $\lambda$/2-plate. Areas of roughly 35 × 5 mm$^{2}$ were textured on the samples for each set of parameters used. All structuring experiments were carried out under ambient conditions to ensure industrial applicability.

The topography of the samples was investigated using a confocal microscope at a 50x magnification with an optical and vertical resolution of 170 nm and 3 nm, respectively. Afterwards, the collected topographic data were evaluated with a surface analyzing software. Profiles were extracted from the topographic data according to ISO 16610^51^.

The specimens were also characterized with a scanning electron microscope at an accelerating voltage of 6.0 kV. The chemical composition of the sample surface was evaluated by energy-dispersive X-ray spectroscopy (EDX) accessory at an accelerating voltage of 15 kV. Additionally, an optical microscope with 50$\times$ objective was used to determine the grain morphology micro-structure of the samples. Before that, the samples were etched at a constant temperature of 50 $^\circ$C for five minutes in a stainless steel stain with hydrochloric acid and nitric acid concentration of 15–20 $\%$ and 1$-<$5 $\%$, respectively.

## Supplementary Information

Supplementary Information.

## Data Availability

The data sets used and/or analyzed during the current study are available from the corresponding author on reasonable request.

## References

[1] Vorobyev AY, Guo C (2013). Direct femtosecond laser surface nano/microstructuring and its applications. Laser Photonics Rev..

[2] Jagdheesh R, García-Ballesteros J, Ocaña J (2016). One-step fabrication of near superhydrophobic aluminum surface by nanosecond laser ablation. App. Surf. Sci..

[3] Stratakis E (2020). Laser engineering of biomimetic surfaces. Mater. Sci. Eng. R. Rep..

[4] Vercillo V (2020). Design rules for laser-treated icephobic metallic surfaces for aeronautic applications. Adv. Func. Mater..

[5] Epperlein N (2017). Influence of femtosecond laser produced nanostructures on biofilm growth on steel. Appl. Surf. Sci..

[6] Guay J-M (2018). Topography tuning for plasmonic color enhancement via picosecond laser bursts. Adv. Opt. Mater..

[7] Lasagni AF (2017). Laser interference patterning methods: Possibilities for high-throughput fabrication of periodic surface patterns. Adv. Opt. Technol..

[8] Fraggelakis F, Tsibidis GD, Stratakis E (2021). Tailoring submicrometer periodic surface structures via ultrashort pulsed direct laser interference patterning. Phys. Rev. B.

[9] Schell F (2022). Fabrication of four-level hierarchical topographies through the combination of lipss and direct laser interference pattering on near-beta titanium alloy. Mater. Lett..

[10] Bieda, M., Beyer, E. & Lasagni, A. F. Direct fabrication of hierarchical microstructures on metals by means of direct laser interference patterning. *J. Eng. Mater. Technol.* **132** (2010).

[11] Zwahr C, Helbig R, Werner C, Lasagni AF (2019). Fabrication of multifunctional titanium surfaces by producing hierarchical surface patterns using laser based ablation methods. Sci. Rep..

[12] Sikora A, Faucon M, Gemini L, Kling R, Mincuzzi G (2022). Lipss and dlip: From hierarchical to mutually interacting, homogeneous, structuring. Appl. Surf. Sci..

[13] Guo Z, Liu W, Su B-L (2011). Superhydrophobic surfaces: From natural to biomimetic to functional. J. Colloid Interface Sci..

[14] Bonse J, Gräf S (2020). Maxwell meets marangoni-a review of theories on laser-induced periodic surface structures. Laser Photon. Rev..

[15] Skolski J, Römer G, Vincenc Obona J, Huis in’t Veld A (2014). Modeling laser-induced periodic surface structures: Finite-difference time-domain feedback simulations. J. Appl. Phys..

[16] Aguilar A, Mauclair C, Faure N, Colombier J-P, Stoian R (2017). In-situ high-resolution visualization of laser-induced periodic nanostructures driven by optical feedback. Sci. Rep..

[17] Rudenko A, Mauclair C, Garrelie F, Stoian R, Colombier J-P (2019). Light absorption by surface nanoholes and nanobumps. Appl. Surf. Sci..

[18] Bonse J, Munz M, Sturm H (2005). Structure formation on the surface of indium phosphide irradiated by femtosecond laser pulses. J. Appl. Phys..

[19] Rudenko A (2020). High-frequency periodic patterns driven by non-radiative fields coupled with marangoni convection instabilities on laser-excited metal surfaces. Acta Mater..

[20] Zhang H (2015). Coherence in ultrafast laser-induced periodic surface structures. Phys. Rev. B.

[21] Lalanne P, Hugonin J-P, Liu H, Wang B (2009). A microscopic view of the electromagnetic properties of sub-$$\lambda$$ metallic surfaces. Surf. Sci. Rep..

[22] Nivas JJ, Amoruso S (2021). Generation of supra-wavelength grooves in femtosecond laser surface structuring of silicon. Nanomaterials.

[23] Nyenhuis F, Michalowski A, L’huillier J (2021). Ultrashort pulse surface melting and smoothing: The impact of pulse spacing on heat accumulation and structure formation. J. Appl. Phys..

[24] Bauer F, Michalowski A, Kiedrowski T, Nolte S (2015). Heat accumulation in ultra-short pulsed scanning laser ablation of metals. Opt. Express.

[25] Fraggelakis F, Mincuzzi G, Lopez J, Manek-Hönninger I, Kling R (2017). Texturing metal surface with mhz ultra-short laser pulses. Opt. Express.

[26] Nyenhuis, F. *et al.* Fundamentals of scanning surface structuring by ultrashort laser pulses: From electron diffusion to final morphology. *Adv. Photonics Res.* 2200045 (2022).

[27] Tsibidis GD, Skoulas E, Papadopoulos A, Stratakis E (2016). Convection roll-driven generation of supra-wavelength periodic surface structures on dielectrics upon irradiation with femtosecond pulsed lasers. Phys. Rev. B.

[28] Neuenschwander, B., Jaeggi, B., Schmid, M., Rouffiange, V. & Martin, P. -E. Optimization of the volume ablation rate for metals at different laser pulse-durations from ps to fs. In *Laser Applications in Microelectronic and Optoelectronic Manufacturing (LAMOM) XVII*, vol. 8243, 824307 (International Society for Optics and Photonics, 2012).

[29] Rudenko A (2017). Spontaneous periodic ordering on the surface and in the bulk of dielectrics irradiated by ultrafast laser: A shared electromagnetic origin. Sci. Rep..

[30] Florian C, Déziel J-L, Kirner SV, Siegel J, Bonse J (2020). The role of the laser-induced oxide layer in the formation of laser-induced periodic surface structures. Nanomaterials.

[31] Zhai Z, Wei C, Zhang Y, Cui Y, Zeng Q (2020). Investigations on the oxidation phenomenon of sic/sic fabricated by high repetition frequency femtosecond laser. Appl. Surf. Sci..

[32] Egerton R, Li P, Malac M (2004). Radiation damage in the tem and sem. Micron.

[33] Akhmanov SA, Emel’yanov VI, Koroteev NI, Seminogov VN (1985). Interaction of powerful laser radiation with the surfaces of semiconductors and metals: Nonlinear optical effects and nonlinear optical diagnostics. Soviet Phys. Uspekhi.

[34] Nolte S (1997). Ablation of metals by ultrashort laser pulses. JOSA B.

[35] Dostovalov A, Korolkov V, Okotrub K, Bronnikov K, Babin S (2018). Oxide composition and period variation of thermochemical lipss on chromium films with different thickness. Opt. Express.

[36] Dostovalov AV (2017). Study of the formation of thermochemical laser-induced periodic surface structures on cr, ti, ni and nicr films under femtosecond irradiation. Quantum Electron..

[37] Dostovalov AV (2019). Lipss on thin metallic films: New insights from multiplicity of laser-excited electromagnetic modes and efficiency of metal oxidation. Appl. Surf. Sci..

[38] Li Z (2009). Analysis of oxide formation induced by uv laser coloration of stainless steel. Appl. Surf. Sci..

[39] Sedao X (2018). Self-arranged periodic nanovoids by ultrafast laser-induced near-field enhancement. ACS Photonics.

[40] Menold T, Ametowobla M, Werner J (2021). Signatures of self-interstitials in laser-melted and regrown silicon. AIP Adv..

[41] Menold T, Hadjixenophontos E, Lawitzki R, Schmitz G, Ametowobla M (2020). Crystal defects in monocrystalline silicon induced by spot laser melting. J. Appl. Phys..

[42] He M, Karim ET, Shugaev MV, Zhigilei LV (2021). Atomistic simulation of the generation of vacancies in rapid crystallization of metals. Acta Mater..

[43] Zhang H, Liu F, Yang Y, Sun D (2017). The molecular dynamics study of vacancy formation during solidification of pure metals. Sci. Rep..

[44] Lin Z, Johnson RA, Zhigilei LV (2008). Computational study of the generation of crystal defects in a bcc metal target irradiated by short laser pulses. Phys. Rev. B.

[45] Zaum C, Osterloh N, Darkins R, Duffy D, Morgenstern K (2021). Real-space observation of surface structuring induced by ultra-fast-laser illumination far below the melting threshold. Sci. Rep..

[46] Nürnberger P (2015). Influence of substrate microcrystallinity on the orientation of laser-induced periodic surface structures. J. Appl. Phys..

[47] Sedao X (2014). Influence of crystal orientation on the formation of femtosecond laser-induced periodic surface structures and lattice defects accumulation. Appl. Phys. Lett..

[48] Yasumaru N, Miyazaki K, Kiuchi J (2005). Fluence dependence of femtosecond-laser-induced nanostructure formed on tin and crn. Appl. Phys. A.

[49] Ränke, F., Baumann, R., Voisiat, B. & Lasagni, A. F. High throughput laser surface micro-structuring of polystyrene by combining direct laser interference patterning with polygon scanner technology. *Materials Letters: X* 100144 (2022).

[50] Schröder N, Vergara G, Voisat B, Lasagni AF (2020). Monitoring the heat accumulation during fabrication of surface micropatterns on metallic surfaces using direct laser interference patterning. J. Laser Micro Nanoeng..

[51] Pomberger S, Stoschka M, Leitner M (2019). Cast surface texture characterisation via areal roughness. Precis. Eng..

