# Supplementary material for: Heating influence on hierarchical structures fabricated by direct laser interference patterning
Source: Sci Rep. 2022 Oct 22;12:17728. doi: 10.1038/s41598-022-22368-w (PMC9588078; doi:10.1038/s41598-022-22368-w)
Supplement: Supplementary file 1 — Supplementary Information. [file 41598_2022_22368_MOESM1_ESM.pdf]

Heating influence on hierarchical structures fabricated by direct laser interference patterning

Nikolai Schröder[1,a*], Fabian Nyenhuis[2,a], Robert Baumann[1], Lucinda Mulko[1], Thomas Kiedrowski[2], Johannes Albert L'huillier[3], and Andrés Fabián Lasagni[1,4]

[1]Institut für Fertigungstechnik, Technische Universität Dresden, George-Bähr-Strasse 3c, 01069, Dresden, Germany
[2]Robert Bosch GmbH, Bosch Research, Postbox 30 02 40, 70442, Stuttgart, Germany.
[3]Photonik-Zentrum Kaiserslautern e.V. and Research Center OPTIMAS, Technische Universität Kaiserslautern, Kohlenhof Strasse 10, 67633, Kaiserslautern, Germany.
[4]Fraunhofer-Institut für Werkstoff- und Strahltechnik (IWS), Winterbergstrasse 28, 01277, Dresden, Germany
[*]nikolai.schroeder@tu-dresden.de
[a]These authors contributed equally to this work.

ABSTRACT

The combination of direct laser interference patterning (DLIP) with laser-induced periodic surface structures (LIPSS) enables the fabrication of functional surfaces reported for a wide spectrum of materials. The process throughput is usually increased by applying higher average laser powers. However, this causes heat accumulation impacting the roughness and shape of produced surface patterns. Consequently, the effect of substrate temperature on the topography of fabricated features requires detailed investigations. In this study, steel surfaces were structured with line-like patterns by ps-DLIP at 532 nm. To investigate the influence of substrate temperature on the resulting topography, a heating plate was used to adjust the temperature. Heating to 250 °C led to a significant reduction of the produced structure depths, from 2.33 μm to 1.06 μm. The reduction is associated with the appearance of a different LIPSS type, depending on the grain orientation of the substrates and laser-induced superficial oxidation. This study revealed a strong effect of substrate temperature, which is also to be expected when heat accumulation effects arise from processing surfaces at high average laser power.

Supplementary material

Influence of structure on the optical appearance

Fig. S1 illustrates an exemplary optical microscopy image of a produced DLIP structure showing the decrease in reflectivity in the areas covered by smaller structures (LSFL-II). Since the laser and the used microscope illumination are in the mid-visible spectrum, the absorption is increased in the corresponding areas.

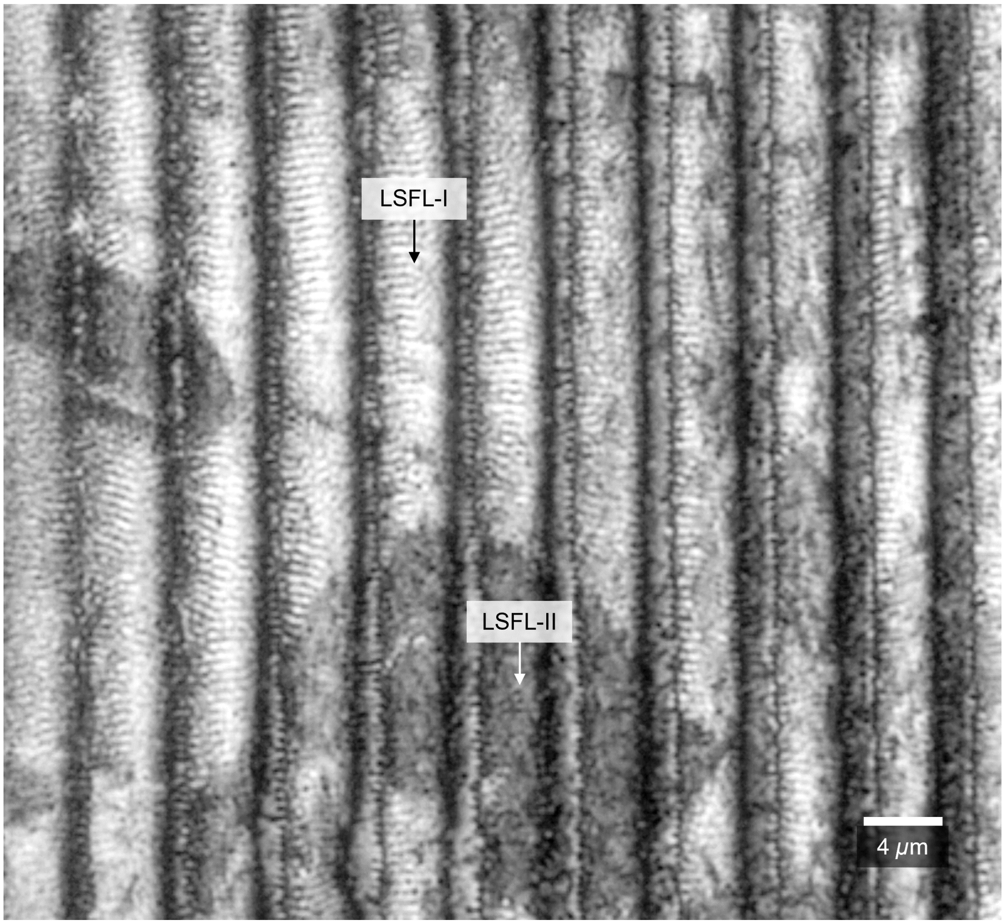

Figure S1. Light microscopy image of the DLIP pattern with a 100x magnification produced at Φ_p = 0.5 J/cm^2 (peak, for Gaussian equivalent without interference), f = 200 kHz, o_p = 99.0 % and T_S = 250 °C. The location of LSFL-I and LSFL-II is highlighted in the image.

Formation of HSFL in the periphery of the intensity profile

LSFL-II need LSFL-II as precursors owing to required intensity pattern. SEM images were taken at the upper edge of the pattern (perpendicular to the scan direction). The images are shown in Fig. S2. For the case of T_S = 250°C, a large amount of HSFL were formed with a higher regularity. Partially, this may be due to changes in crystal defect dynamics discussed in the main manuscript. The HSFL observed at the edge are also expected in the scan direction and consequently can contribute to LSFL-II formation.

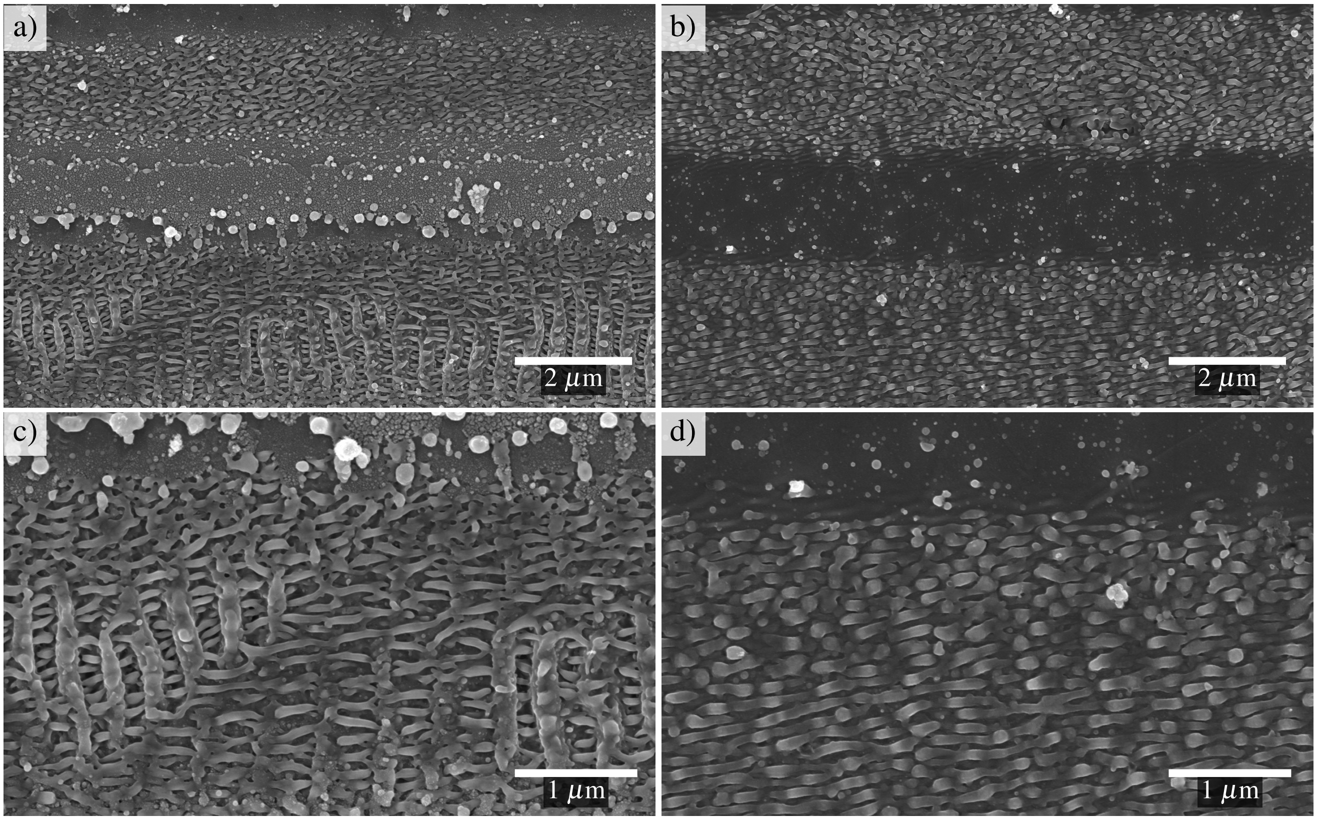

Figure S2. SEM images of the two outer lines in the DLIP pattern produced at Φ_p = 0.5 J/cm^2 (peak, for Gaussian equivalent without interference), f = 200 kHz, o_p = 99.0 % and (a,c) T_S = 21 °C and (b,d) T_S = 250 °C. The outer line features of the pattern experience the lowest fluence since the intensity profile of the interference pattern decreases over distance similarly to its input intensity distributions (in this case Gaussian intensity distributions). Consequently, HSFL occur in the edge regions of the intensity profile, which are also to be expected in the scan direction due to the symmetry of the profile. In the case of increased temperature, only HSFL are observed, which appear more regular than in the case of T_S = 21°C. Furthermore, some LSFL-I already occur at this temperature.

Grain dependence clustered occurrence of LSFL-II

To clarify the cluster-wise LSFL-II appearance, an unprocessed specimen was etched to reveal the grain formation. The grains are compared in Fig. S3 with the dark areas of the SEM images, which result from the transition from LSFL to LSFL-II. As Fig. S3 a) shows, the geometry and size of the areas with different SEM gray values correspond to the dimensions of the grains visible in Fig. S3 b). The change in gray values is associated with a change from LSFL-I to LSFL-II as shown in Fig. **??**. The etching process increased the contrast of the grain boundaries, which appear as dark outlines. The geometry and dimensions of the grains match well with the darker areas in the SEM images.

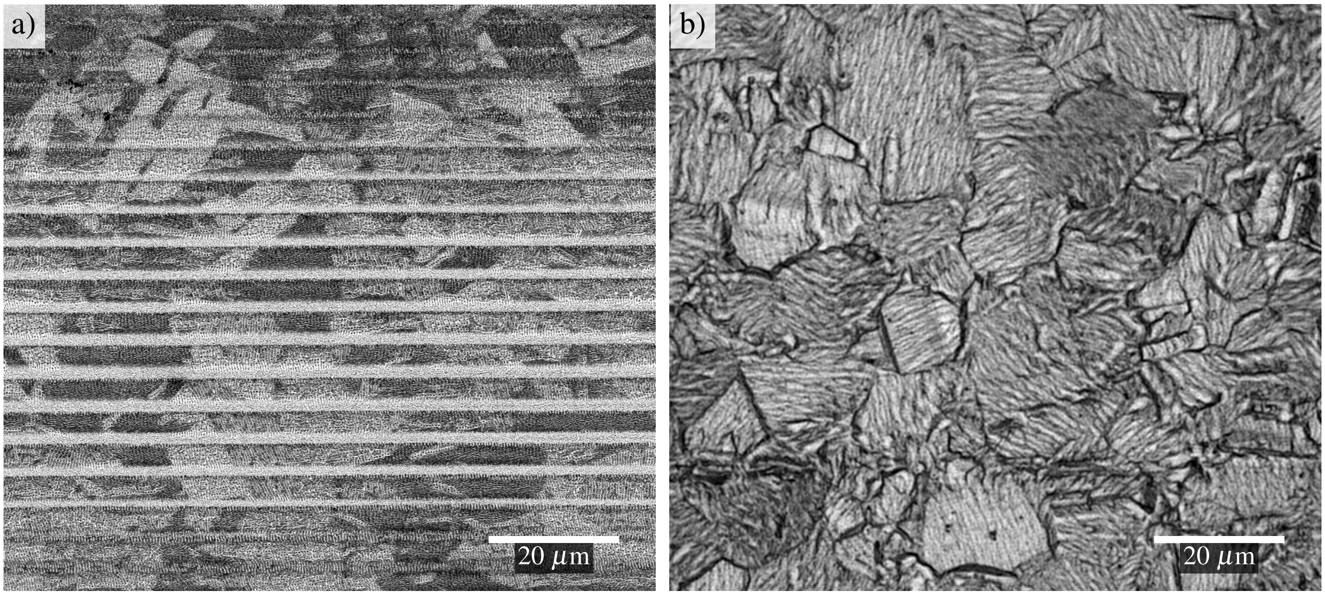

Figure S3. a) SEM image of a surface processed using the DLIP technique (Φ_p = 0.5 J/cm^2 (peak, for Gaussian equivalent without interference), T_S = 250 °C, o_p = 99.0 %, f = 200 kHz). The dark areas exhibit LSFL-II, as shown in Fig. 2 in the main text. In b) light microscope image of an etched, unprocessed sample are shown.
